# Juvenile psammomatoid ossifying fibroma of the posterior fossa: a case report and review

**DOI:** 10.1186/s40064-016-2758-4

**Published:** 2016-07-15

**Authors:** Carlos Cotúa Quintero, Anwar Saab Mazzei, Juan Revuelta Barbero, Avelino Parajon Diaz, Luis Ley Urzaiz

**Affiliations:** Department of Neurosurgery, Puerta de Hierro University Hospital, Manuel de Falla Street, N° 1, Majadahonda, 28222 Madrid, Spain; Department of Neurosurgery, Ramon and Cajal University Hospital, Colmenar Viejo Street, Km. 9,100, 28034 Madrid, Spain; Oporto N° 1, Portal 3, Floor 1, Apartment A, Pozuelo de Alarcon, PC, 28224 Madrid, Spain

**Keywords:** Fibroma, Ossifying, Psammoma, Occipital, Tumor

## Abstract

**Background:**

Ossifying fibroma is a rare benign bone tumor that occurs mostly in the jaw, but also affects paranasal sinuses and fronto-ethmoidal complex. Occipital bone is an extremely rare location for these tumors; only two cases have been reported.

**Methods:**

We present the first case reported as psammomatoid subtype of ossifying fibroma, according to the 2005 WHO classification. An 18 years old male patient with infratentorial tumor, in the occipital bone, that produces mass effect over the cerebellum.

**Conclusions:**

This case may provide a guide to consider these lesions for a more rapid and precise diagnostic in future cases.

## Introduction

Juvenile psammomatoid ossifying fibroma (JPOF) is a rare benign bone tumor that often affects facial bones, especially the mandible, it commonly occurs in young patients, and the treatment is indicated because of deformity and esthetical reasons mostly, en bloc resection is the treatment of choice. It is as subtype of juvenile ossifying fibroma in the WHO classification of odontogenic tumors (Barnes et al. 2005). JPOF of cranial bones are extremely rare, and, to our knowledge, only two cases affecting the occipital bone have been described; this is the first case reported as a psammomatoid subtype.

## Case report

**Fig. 1:**
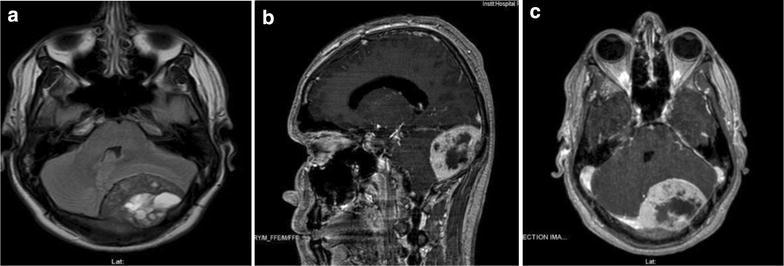
Preoperative MRI images. **a** FLAIR axial, shows an infratentorial tumor that deforms the fourth ventricle, with hiperintense areas that suggest hemorrhage. **b**, **c** T1 with gadolinium enhanced axial and sagital, showing irregular enchantment of the tumor

We present a case of an 18 years old male, with no personal history of interest; he referred 6 weeks of headache and blurred vision. At physical examination he presented right VI nerve palsy with diplopia, and bilateral papiledema. A CT scan reported a left parietooccipital enhancing lesion measuring 6 × 4 cm. The MRI showed a well-defined occipital infratentorial diploe osteolytic lesion, affecting the inner table, hyperintense on T1WI suggesting hemorrhagic areas producing mass effect over the left cerebellum hemisphere and obliteration of the cisterna magna ( Fig. 1).

**Fig. 2:**
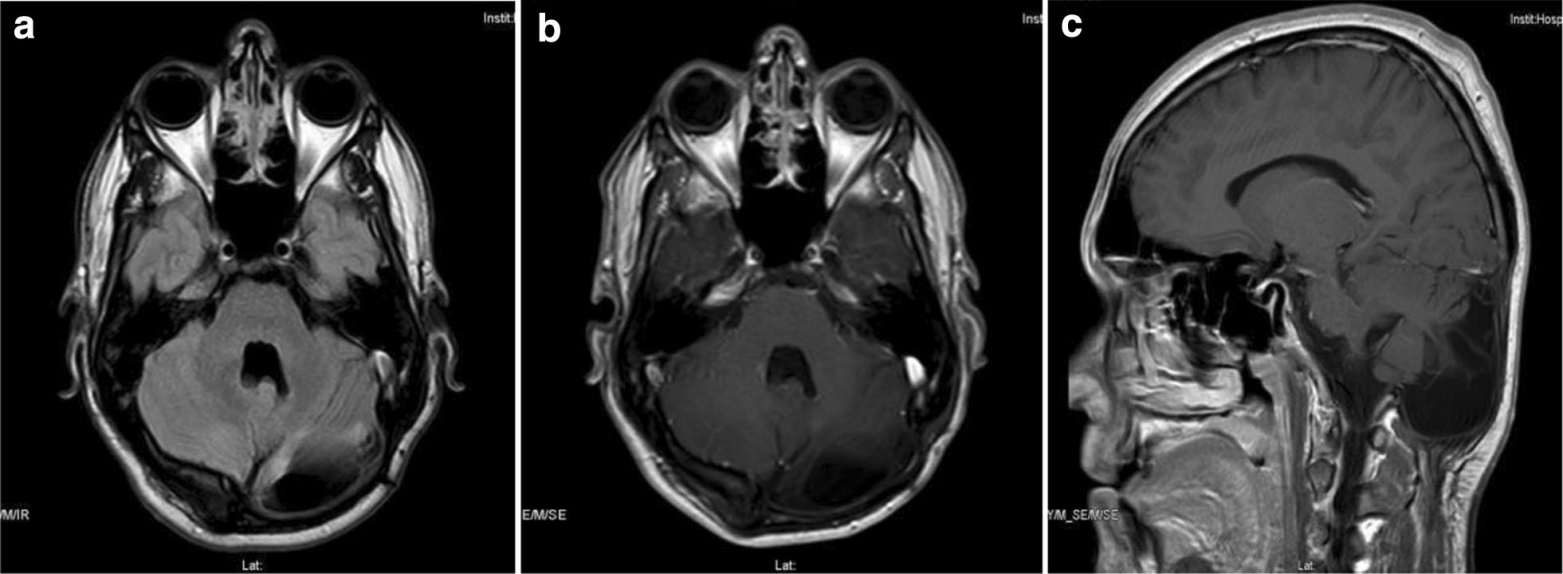
Postoperative MRI images. **a** FLAIR. **b**, **c** T1 gadolinium enhanced: shows complete resection of the tumor with no recurrence after 5 years follow up

After embolization of the occipital artery, surgery was performed, with total resection of the lesion, and the dura also, with subsequent repair, because it was infiltrated. During postoperative care, the patient presented CSF fistula complicated with bacterial meningitis which after antibiotic treatment, and the dura was surgically repaired again. Patient was discharged without any neurological alteration. At 4 years of clinical follow up and annual MRI studies, he remains asymptomatic, carries a normal life and shows no evidence of radiological recurrence (Fig. 2).

## Discussion

**Fig. 3:**
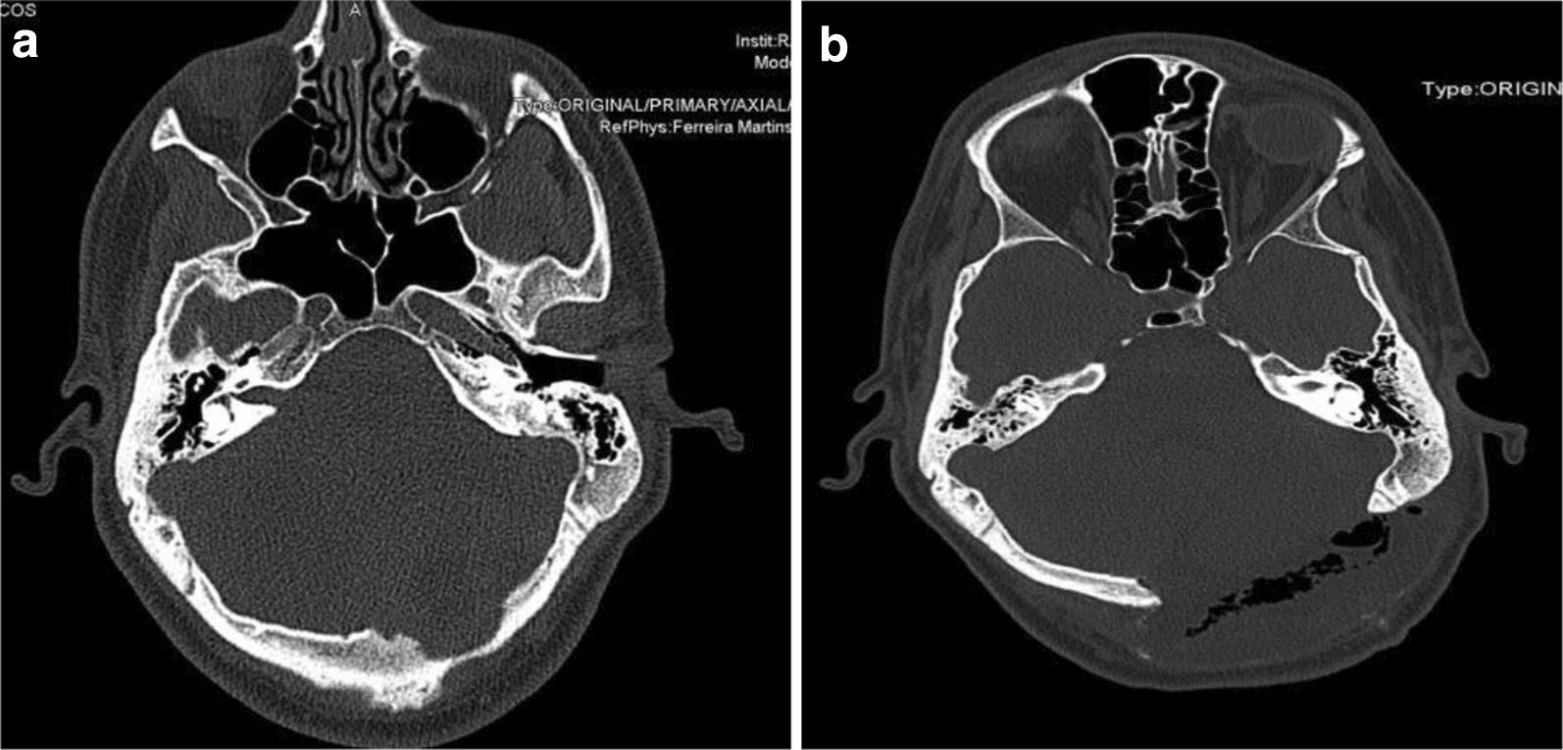
**a** Preoperative CT scan showing an osteolityc lesion in the occipital bone. **b** Postoperative CT scan showing bone resection

JPOF is a fibro-osseous lesion, which generally occurs in the mandible, but can affect any facial bone. Trauma is commonly hypothesized to be the initiating feature of these lesions (Jung et al. 1999). JPOF originates from overproduction of the myxofibrous cellular stroma normally involved in the growth of the septa in the paranasal sinuses as they enlarge and pneumatize (Johnson et al. 1991) (Fig. 3).

**Fig. 4:**
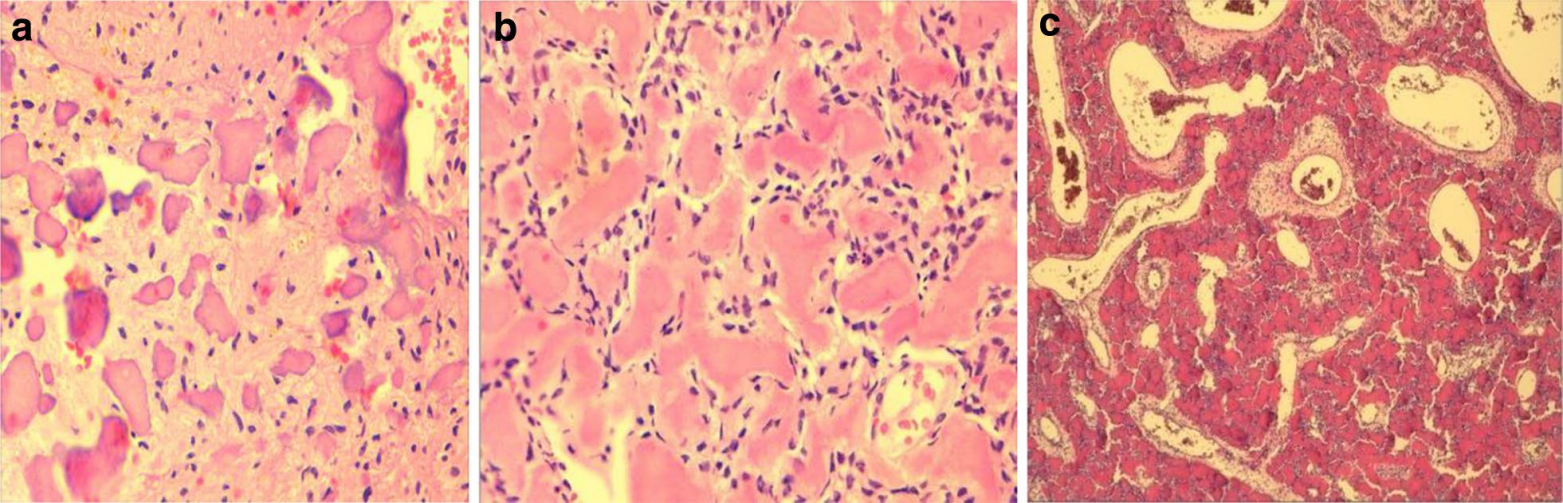
**a**–**c** Photomicrograph with hematoxylin-eosin stain: showing extensive osteoid production in a cellular fibrous stroma, with irregular pseudovascular spaces and round basophilic calcifications

Histologically it is characterized by the presence of concentric or laminated ossicles called psammoma bodies which contain osteocytes that indicate osteogenic origin. These ossicles vary from small with a round to oval shape, to large and irregular shape. They have a basophilic center and a peripheral pink rim that usually shows radiating fibers, thus assuming the appearance of a fringe (Slootweg et al. 1994). The non-osseous component includes cellular proliferation of uniform cytomorphologically bland plump fibroblastic spindle cells and atypical mitosis is absent. It also shows osteoblastic and osteoclastic activity at the periphery of the lesion (Wenig et al. 1995). Mitotic figures are present in cellular stroma, but atypical mitosis is absent (Slootweg et al. 1994) (Fig. 4).

**Fig. 5:**
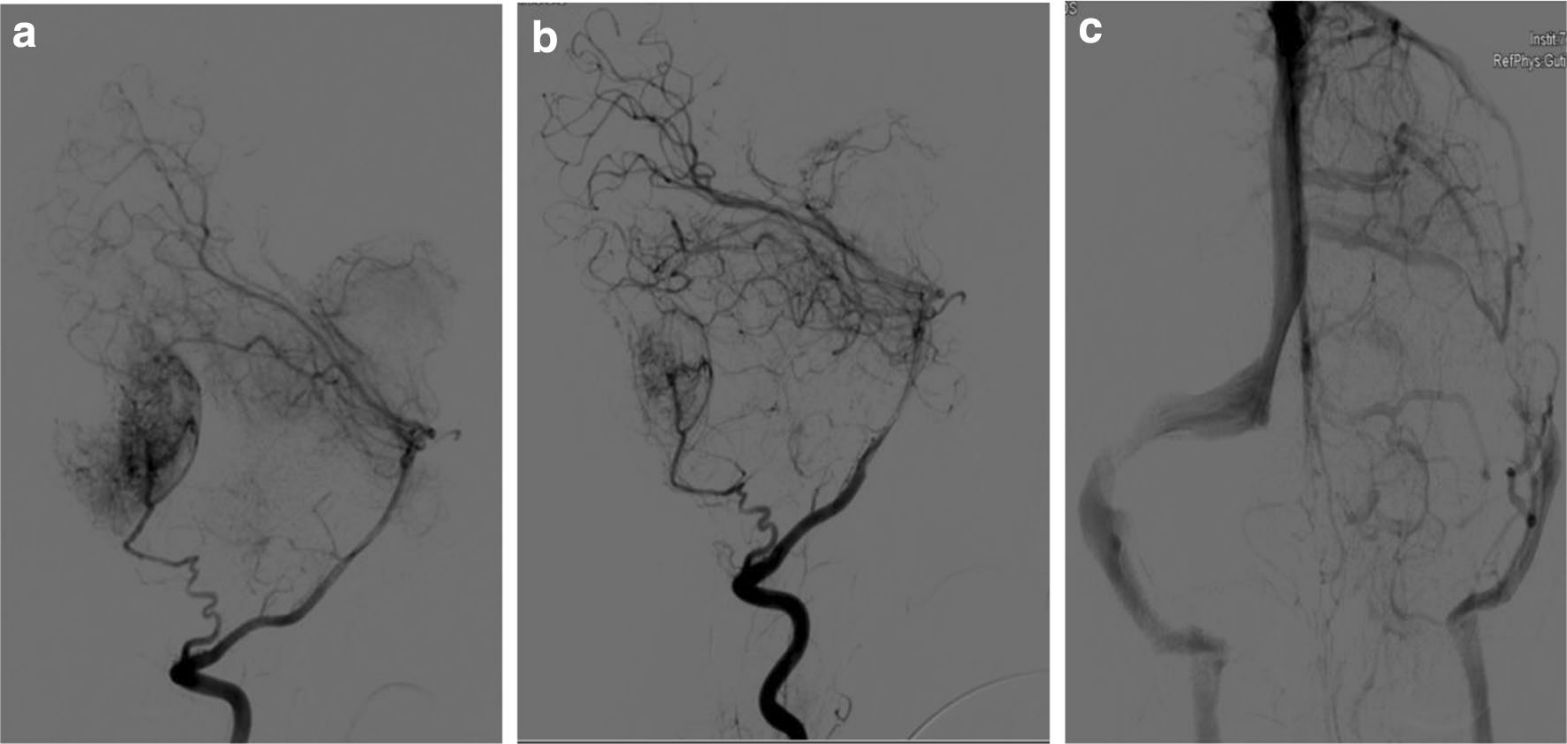
**a** Preembolization arteriography showing a tumor, irrigated by the occipital artery, in the occipital bone. **b** Posembolization arteriography, showing a partially embolization of the tumor. **c** Left transverse sinus compressed by the tumor

Radiographically, these lesions are often expansive and destructive. The most characteristic radiographic feature is an intradiploic lesion that has sharp delimitation from adjacent structures with the usual radiological characteristics as the lesion consisted of both lytic and blastic parts, destroying outer and inner tables. On radiographs, JPOF appears as a monostotic, round or ovoid, well-demarcated, expanding lesion, it can range from predominantly cystic to primarily sclerotic (Chung et al. 2008). On CT scan, JPOF demonstrates a thick shell of bone density outlining the lesion with irregular internal and smooth external surfaces. It has a multiloculated internal appearance and content of varying density (Johnson et al. 1991). On MR images, the solid component usually is isointense relative to muscle on T1-weighted images and on T2-weighted images (Chung et al. 2008). In our case, it shows hiperintesity areas, which suggest hemorrhagic small areas (Chung et al. 2008). After administration of gadolinium contrast material, solid components of the lesion exhibit heterogeneous but diffuse enhancement, where as cystic areas show thin peripheral and septal enhancement (Chung et al. 2008). Angiography, reveals irrigation by extracerebral vessels, although may be avascular cases (Fig. 5).

JPOF generally occurs in a young age (Johnson et al. 1991; Makek 1983; Margo et al. 1985) with a mean of 17.7 years (Makek 1983), the most affected site are the paranasal sinuses (61.6–70 %), where the commonest are the ethmoidal sinus, followed by maxilla (20 %) and mandible (7–10 %). It is infrequent in the calvaria (10–12 %) (Johnson et al. 1991; Makek 1983), affecting most commonly the parietal bone (4.6 %), temporal bone 3.5 % and frontal bone 3.4 %. (Makek 1983).

The main differential diagnosis is fibrous dysplasia. JPOF usually is monostotic and well demarcated lesion, lacks ground-glass attenuation, and contains areas of mineralization (Makek 1983; Smith and Zabaleta 1952; Wenig et al. 1995; Yamashita et al. 1977). In addition, fibrous dysplasia tends to be more elongated because it is a developmental abnormality rather than a neoplastic process (Chung et al. 2008; Johnson et al. 1991). Histologically, JPOF is a fibroosseous proliferation composed of fibrous stroma, admixed bony spicules, and the presence of psammomatoid ossicles. In contrast to fibrous dysplasia, osteoclasts and osteoblasts typically line the trabeculae, which are composed of entrapped lamellar bone.

Other differential diagnosis is to be made with conventional ossifying fibroma (COF) and with juvenile trabecular ossifying fibroma (JTOF). COF is an odontogenic neoplasm arising from the periodontal ligament and affecting the tooth bearing are as of the mandible, and maxilla, whereas the nasoethmoidal location is rare (Speight and Carlos 2006). COF is well-encapsulated and radiologically presents as an expansile soft-tissue-mass with sharp demarcation from adjacent bone. On the contrary, the juvenile variants, JPOF and JTOF are unencapsulated and arise outside of the tooth bearing areas, either in the maxilla or the craniofacial skeleton and affect a younger group of age where as the juvenile trabecular fibroma appears in patients below 15 years and both with slightly predilection to male patients (El-Mofty 2002). JPOF commonly affects patients older than JTOF. Histologically, the juvenile variants share a similar stroma but JPOF is characterized by innumerable small ossicles resembling psammoma bodies, while JTOF contains trabeculae of fibrillary osteoid and woven bone (El-Mofty 2002; Speight and Carlos 2006).

Only two cases of ossifying fibroma in the occipital bone have been described in the literature. Yamashita et al. published the first case back in 1977; the second case is published by Ozcan et al. in 1995; both of them in asymptomatic patients and without a subtype classification. We present the first case of occipital JPOF with neurological manifestations (Table 1).

**Table 1:** Cases of JPOF of the occipital bone

Case	Year	Gender	Age	Neurological deficit	Treatment	Symptoms	Subtype
Yamashita	1977	Female	13	No	Surgery	Local swelling	Not described
Ozcan	1995	Male	9	No	Surgery	Local swelling	Not described
Present case	2015	Male	18	Yes	Surgery	Blurred vision, VI nerve palsy	Psammomatoid

The treatment of choice for JPOF is complete surgical excision. Partial or incomplete resection leads to recurrences and is reported to range from 30 to 56 % (Bertrand et al. 1993; Margo et al. 1985). In our case, a total in bloc resection was performed, with no recurrence after 5 years follow up. Radiotherapy is not indicated because of its radio resistance and post-radiation complications (Jung et al. 1999). Malignant degeneration has not been reported so far.

## Conclusion

The JPOF is a benign tumor with aggressive behavior that rarely affects the cranial bones; this is the first reported case within this localization causing neurological deficits. Total resection is the treatment of choice. Prognosis is good, without any case of metastasis being reported.
